# Aerobic fitness and body composition independently relate to inhibitory control in preadolescent children

**DOI:** 10.1038/s41598-026-63026-9

**Published:** 2026-07-18

**Authors:** Kellyn E. Carlson, Christina M. Bertrand, Lauren B. Raine, Jennifer N. H. Watrous, Charles H. Hillman

**Affiliations:** 1 Department of Physical Therapy, Movement, & Rehabilitation Sciences, Northeastern University, Boston, MA USA; 2 Institute for Cognitive and Brain Health, Northeastern University, Boston, MA USA; 3 Department of Medical Sciences, Northeastern University, Boston, MA USA; 4 Department of Psychology, Oklahoma State University, Stillwater, OK USA; 5 Department of Psychology, Northeastern University, Boston, MA USA

**Keywords:** Inhibition, Event-related potentials, Neurocognitive development, Physical activity, Body fat percent, Neuroscience, Psychology, Psychology

## Abstract

Aerobic fitness and body composition are modifiable factors that independently relate to inhibitory control, with higher aerobic fitness associated with enhanced performance and higher body fat associated with poorer inhibitory control in children. This study examined these factors as independent predictors of behavioral and neuroelectric outcomes during a Go/NoGo task. One hundred and two preadolescent children (mean age: 10.07 ± 0.67 years) completed VO_2_peak assessments and DXA scans before performing a Go/NoGo task while ERPs were recorded. Linear regressions and mixed-effects models examined main and interaction effects of fat-free aerobic fitness (FFVO_2_peak) and body fat percent on behavior, N2 and P3 amplitude and latency across seven midline electrode sites. Higher aerobic fitness was associated with greater NoGo accuracy, while body composition was unrelated to behavioral performance. Aerobic fitness showed topographic interactions with N2 and P3 amplitude during the Go task and with N2 amplitude during the NoGo task, reflecting greater neural resource allocation at fronto-central and centro-parietal sites. Body composition showed a topographic interaction with NoGo P3 latency, with higher body fat associated with shorter latencies. These findings demonstrate dissociable topographic effects of aerobic fitness and body composition on inhibitory control, suggesting distinct pathways of neurocognitive influence in preadolescent children.

**Supplementary Information:**

The online version contains supplementary material available at https://doi.org/10.1038/s41598-026-63026-9.

## Introduction

Childhood physical inactivity represents a critical public health concern in the industrialized world. According to the 2024 U.S. Report Card on Physical Activity for Children and Youth, just one in four children meets the recommended 60 min per day of moderate-to-vigorous physical activity^1^. Such inactivity poses a substantial public health burden, contributing to an estimated $117 billion in annual health care costs^2^. Compounding this, roughly 25% of children in the U.S. are obese or severely obese^3^, which is associated with roughly $7.5 billion annually in aggregate medical care^4^. Beyond physical health consequences, poorer aerobic fitness and less healthy body composition in childhood have been associated with compromised cognitive and neural processing, including executive functions (a subset of cognitive processes involving the intentional component of environmental interaction, including inhibition, working memory, and mental flexibility) that support academic achievement^5–9^. Understanding the independent contributions of fitness and body composition to cognitive function is critical for developing targeted interventions to support both physical and cognitive health in children. Distinguishing their independent contributions is important because fitness and body composition are modifiable through different behavioral and lifestyle factors. Understanding whether each factor uniquely predicts cognitive outcomes may clarify the distinct mechanisms at play, which will guide the development of more precise and targeted interventions.

A growing body of evidence has demonstrated that childhood aerobic fitness is positively associated with cognitive function, especially performance on behavioral tasks measuring executive functions^6,9–15^. A 2016 systematic review by Donnelly, Hillman, and colleagues synthesized 18 studies examining associations between physical activity, aerobic fitness, and brain function^16^. Several of these studies examined neural correlates of fitness-related cognitive differences using event-related potentials (ERPs), which reflect time-locked neuroelectric (i.e., electroencephalographic [EEG]) activity in response to a stimulus or action^17^. This line of research highlighted that higher aerobic fitness in preadolescent children is related to enhanced neuroelectric resources supporting attention, inhibition, cognitive flexibility, and conflict monitoring/error detection^16^. These findings indicate that aerobic fitness plays a critical role in both behavioral and neuroelectric indices of executive function. Still, aerobic fitness operates in conjunction with other demographic and health factors including body composition (e.g., body mass index [BMI], adiposity), which is another modifiable factor that may independently influence cognitive performance. To account for the relationship between body composition and aerobic fitness, the present study operationalized fitness as VO_2_peak normalized to lean body mass (FFVO_2_peak), allowing for a more independent examination of fitness effects on cognitive performance.

Such an approach is important, because body composition has also been found to influence cognitive and brain outcomes^6,18,19^. For example, an ERP investigation of inhibitory control found that children with obesity demonstrated lower response accuracy and a different topographic distribution than their healthy-weight peers^6^. In a subsequent study examining the acute effects of physical activity, children performed better on an inhibitory control task after a walking intervention compared to seated rest, but this effect was blunted in children with higher BMI^18^. Additionally, magnetic resonance imaging research has shown that deviation from normal BMI is related to reduced mean cortical thickness, specifically within prefrontal cortical regions, as well as worse cognitive task performance compared to normal-weight peers^19^. Together, these findings suggest that higher BMI is associated with poorer behavioral performance and neuroelectric variability in executive functioning in children. Though existing studies have largely relied on BMI as a proxy for body composition, BMI does not distinguish between fat mass and lean mass, limiting its precision as a measure of adiposity. The present study moves beyond BMI by using dual-energy X-ray absorptiometry (DXA) to assess whole-body fat percentage, providing a more direct and precise measure of adiposity. Although existing research has examined fitness and body composition in isolation, it remains an open empirical question whether their contributions to inhibitory control are shared or independent. Thus, examining their unique effects on inhibitory control is essential for clarifying whether these factors are associated with similar or dissociable neurocognitive and behavioral processes.

Inhibitory control, an executive function that supports goal-directed behavior, refers to the ability to regulate and suppress irrelevant responses, stimuli, and memories to maintain focus^20^. Inhibition can be measured by behavioral performance (e.g., response accuracy) on various experimental paradigms including the Go/NoGo task^21^, in which NoGo trials require participants to upregulate inhibitory control by withholding a prepotent response. Because cognitive and automatic systems continue to develop throughout childhood and adolescence^22^, and both aerobic fitness and body composition undergo significant developmental changes during this period^23,24^, preadolescence is a crucial period for understanding how individual differences in physical health may relate to inhibitory control. In addition to behavioral measures, neuroelectric measures such as those derived from ERPs provide temporally precise indices of inhibitory processing. Two primary ERP components associated with inhibitory control include the N2 and P3^25,26^, both of which are typically upregulated during NoGo conditions that require greater amounts of response inhibition compared to Go conditions^27–29^.

The N2 is a negative-going component that occurs roughly 200–400 ms after stimulus onset in the frontocentral scalp topographic region and is typically larger during NoGo, relative to Go, trials^30,31^. The N2 component reflects inhibitory neural processes^28,32^ and conflict monitoring^33–35^. In addition to amplitude, N2 latency indexes the speed of inhibitory processing, with longer latencies indicating slower or less efficient neural inhibition^30^. While some studies have not found a relationship between aerobic fitness and N2 during cognitive tasks^36,37^, it has been hypothesized that the relationship between fitness and inhibition is specific to situations that produce increased conflict^37^. Body composition has also been associated with N2 amplitude, with prior work showing that healthy-weight children exhibit greater N2 amplitudes compared to children with obesity during the NoGo task^6,38^.

The P3 component occurs approximately 300 to 700 ms after stimulus onset and is typically largest over frontoparietal scalp regions during the NoGo task, and over parietal scalp regions during the Go task^27,29,39^. P3 amplitude reflects the allocation of attentional resources, with higher amplitude indicating that more neural resources are activated^25^. Additionally, P3 latency is considered an index of stimulus evaluation speed, such that longer latencies reflect slower cognitive processing during inhibitory demands^25^. Hillman et al.^36^ and Hillman et al.^11^ also demonstrated that higher-fit children had greater P3 amplitudes compared to lower-fit children during tasks requiring executive control. Indeed, Raine and colleagues^8^ conducted a large-scale reanalysis of childhood fitness and inhibitory control and found fitness positively related to larger P3 amplitudes during flanker incongruent trials requiring the upregulation of inhibitory control. Weight status has also been associated with differences in P3 topography, with healthy-weight children demonstrating a more anterior topographic distribution during NoGo trials than their peers with obesity, which may indicate less efficient attentional allocation in children with obesity^6,38^.

### Present study

Despite the growing body of evidence linking aerobic fitness and body composition to cognitive function, few studies have systematically examined their independent contributions to inhibitory control and its neural correlates. The present study addressed this gap by examining ERP indices and behavioral task performance during a Go/NoGo task among preadolescent children with varying fitness and body composition. We hypothesized that higher aerobic fitness would be associated with superior behavioral performance and enhanced neural processing (i.e., larger N2 and P3 amplitudes) during an inhibitory control task, independent of body composition. We further hypothesized that greater whole body fat percentage would be associated with poorer behavioral performance and less efficient neural processing, independent of aerobic fitness. Finally, we hypothesized that each factor would show site-specific interactions with N2 and P3 amplitude and latency, with fitness and body composition potentially showing distinct topographic effects on neuroelectric indices of inhibitory control. By isolating these factors, this research informs more precise recommendations for supporting neurocognitive health during this critical developmental period, when executive functions and related neural resources are rapidly maturing.

## Results

### Task behaviors

Descriptive statistics for task performance were as follows: Go task accuracy (*M* = 96.55%, *SD* = 5.74), Go task RT (*M* = 496.17 ms, *SD* = 62.98), and Go task SDRT (*M* = 99.84 ms, *SD* = 24.83); NoGo task accuracy (*M* = 92.50%, *SD* = 8.66). Linear regression models controlling for sex, pubertal status, and mother’s education, with fitness and body composition entered simultaneously to examine their independent contributions, revealed no significant associations between fitness and Go task accuracy (β = 0.05; 95% CI [-0.10, 0.16], *p =* .637), Go task RT (β = 0.004; 95% CI [-1.08, 1.62], *p =* .691) or Go task SDRT (β = 0.04; 95% CI [-0.61, 0.42], *p =* .714). Body composition was similarly not associated with Go task accuracy (β = 0.16; 95% CI [-0.05, 0.35], *p =* .145) or Go task SDRT (β = -0.005; 95% CI [-0.78, 0.82], *p =* .963).

Higher fitness was independently associated with greater NoGo accuracy (β = 0.26; 95% CI [0.06, 0.45], *p =* .013), after controlling for the same covariates and body composition. Body composition was not independently associated with NoGo accuracy (β = 0.05; 95% CI [-0.23, 0.38], *p =* .635) after accounting for fitness.

### Go task

#### N2 Amplitude

**Fig. 1:**
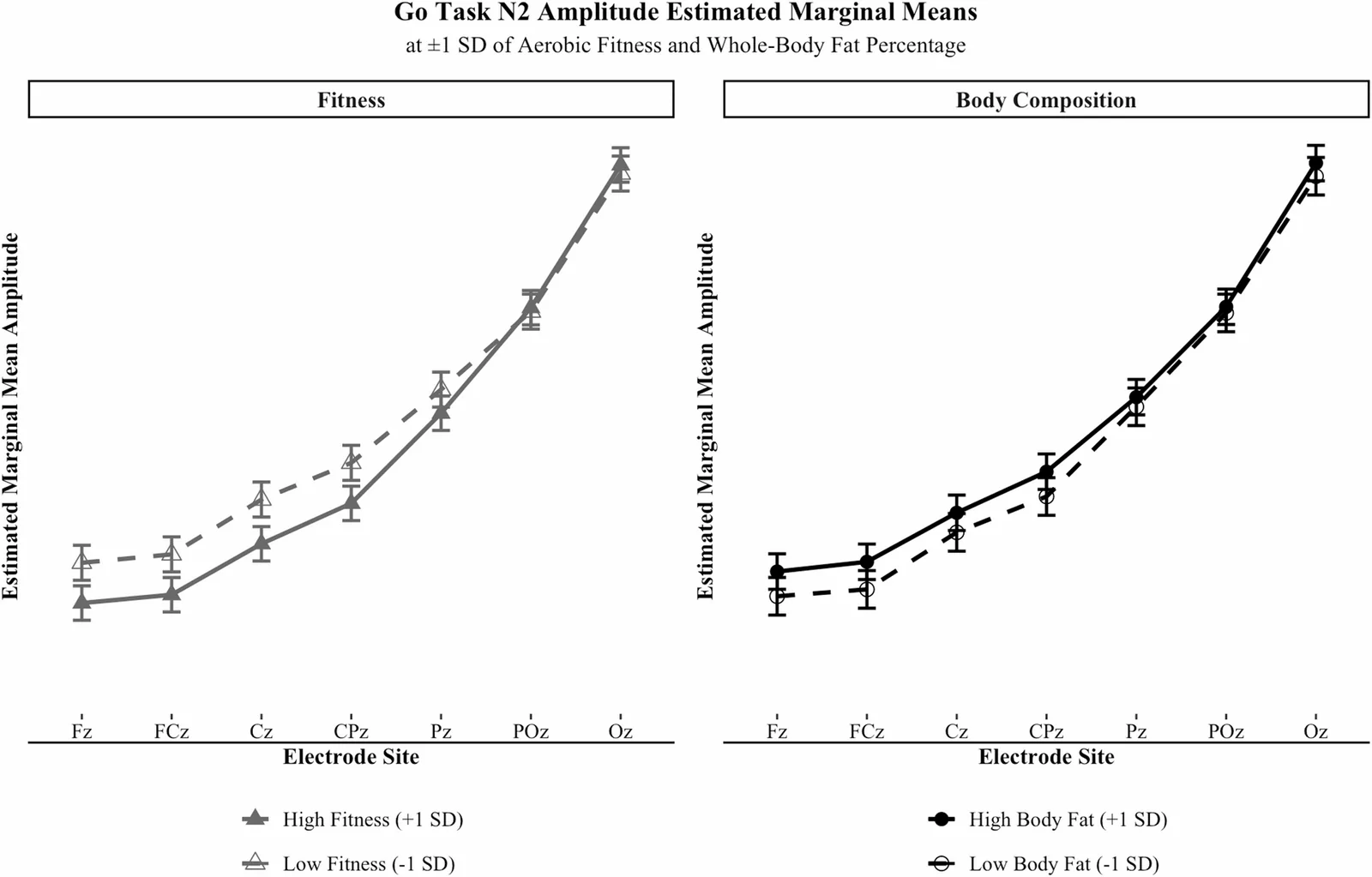
Go Task N2 Amplitude Estimated Marginal Means at ± 1 SD of Aerobic Fitness and Whole-Body Fat Percentage Across Seven Midline Electrode Sites. *Note*. A significant Fitness × Electrode Site interaction was observed, *F (*6, 600) = 2.34, *p =* .030, reflecting that the association between fitness and N2 amplitude varied across the scalp. Higher fitness was associated with more negative N2 amplitude at fronto-central sites and less negative N2 at parietal–occipital sites, as shown in the estimated marginal means at +/-1 SD of fitness. However, simple slopes of fitness at individual electrode sites did not reach significance (all *p’s* > 0.05), suggesting that this interaction reflects a modest, distributed topographic pattern rather than a strong focal effect at any single site. Estimated marginal means are adjusted for sex, pubertal status, and mother’s education. Error bars represent ± 1 SE. *p < .05, ** *p <* .01, *** *p <* .001.

The linear mixed-effects model revealed no main effect of Fitness on N2 amplitude, *F (*1, 96) = 1.57, *p =* .213; however as expected, there was a main effect of Electrode Site, *F (*6, 600) = 425.41, *p <* .001. The Fitness x Electrode Site interaction was also significant, *F (*6, 600) = 2.34, *p =* .030. Probing of EMMs revealed a descriptive topographic pattern, where higher fitness was associated with more negative N2 amplitudes at fronto-central sites (Fz, FCz, Cz, CPz), less negative amplitudes over parietal sites (Pz, POz), and positive-going amplitudes at Oz (see Table 1). Consistent with this, post hoc probing of simple slopes indicated that no individual electrode sites reached significance (all p’s > 0.05), suggesting the interaction reflects a distributed topographic pattern rather than a focal effect at any single site (see Fig. 1).

**Table 1:** N2 Amplitude EMMs by fitness.

Task	Site	Fitness + 1 SD			Fitness − 1 SD		
		95% CI	Mean	SE	95% CI	SD Mean	SE
Go	Fz	-34.65, -28.63	-31.64	1.53	-31.13, -25.01	-28.07	1.55
	FCz	-33.91, -27.89	-30.90	1.53	-30.38, -24.26	-27.32	1.55
	Cz	-29.39, -23.37	-26.38	1.53	-25.52, -19.40	-22.46	1.55
	CPz	-25.81, -19.79	-22.80	1.53	-22.27, -16.14	-19.21	1.55
	Pz	-17.82, -11.80	-14.81	1.53	-15.77, -9.65	-12.71	1.55
	POz	-8.46, -2.44	-5.45	1.53	-8.86, -2.73	-5.79	1.55
	Oz	4.20, 10.22	7.21	1.53	3.38, 9.51	6.44	1.55
NoGo	Fz	-31.02, -24.82	-27.92	1.57	-26.38, -20.07	-23.22	1.60
	FCz	-30.49, -24.29	-27.39	1.57	-25.99, -19.68	-22.84	1.60
	Cz	-27.22, -21.02	-24.12	1.57	-22.64, -13.76	-19.48	1.60
	CPz	-23.89, -17.69	-20.79	1.57	-14.57, -8.26	-16.91	1.60
	Pz	-16.53, -10.33	-13.43	1.57	-14.57, -8.26	-11.42	1.60
	POz	-8.94, -2.71	-5.84	1.57	-7.60, -1.29	-4.44	1.60
	Oz	3.45, 9.66	6.55	1.57	2.77, 9.08	5.92	1.60

Note. Estimated marginal means are shown at -1 SD (60.66 (mL/kg FFM/min) and + 1 SD (79.11 (mL/kg FFM/min) of fat-free peak oxygen consumption.

There was no main effect of Body Composition on N2 amplitude, *F (*1, 96) = 0.70, *p =* .404, but there was the expected main effect of Electrode Site, *F (*6, 600) = 24.12, *p <* .001. The interaction between Body Composition and Electrode Site was not significant, *F (*6, 600) = 0.30, *p =* .939.

#### N2 Latency

There was no main effect of Fitness on N2 latency, *F (*1, 96) = 0.04, *p =* .850; however as expected, there was a main effect of Electrode Site, *F (*6, 600) = 6.14, *p <* .001. The Fitness x Electrode Site interaction was not significant, *F (*6, 600) = 0.45, *p =* .842.

There was also no main effect of Body Composition *F (*1, 96) = 1.10, *p =* .296 nor of Electrode Site, *F (*6, 600) = 0.70, *p =* .654 on N2 latency. The interaction between Body Composition x Electrode Site was also non-significant, *F (*6, 600) = 0.65, *p =* .694.

#### P3 Amplitude

**Fig. 2:**
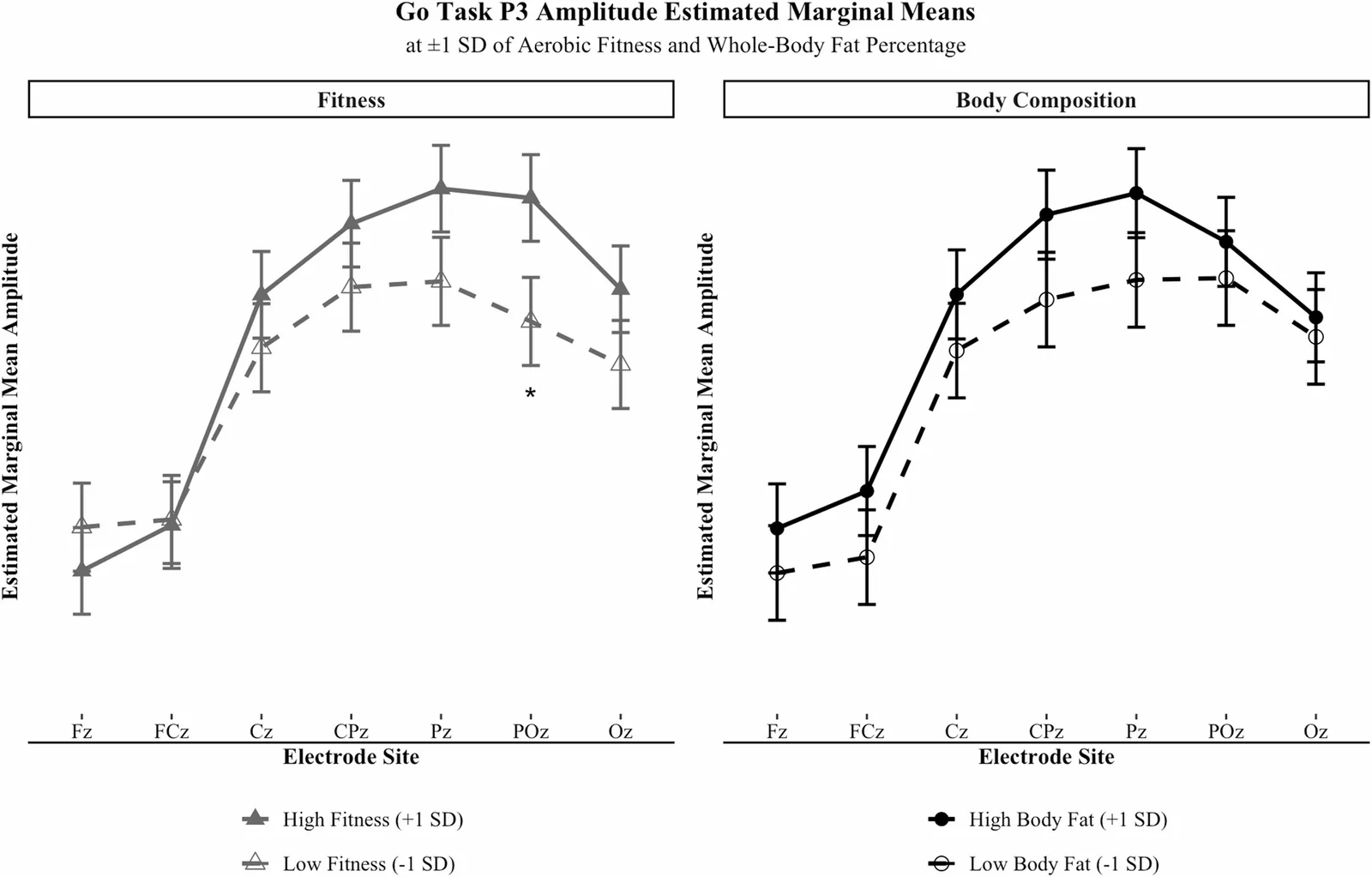
Go Task P3 Amplitude Estimated Marginal Means at ± 1 SD of Aerobic Fitness and Whole-Body Fat Percentage Across Seven Midline Electrode Sites. *Note.* A significant Fitness × Electrode Site interaction was observed, *F (*6, 600) = 2.91, *p =* .008, indicating that the association between fitness and P3 amplitude differed across midline sites. Higher fitness tended to be associated with larger P3 amplitudes at central–parietal sites, with the simple slope of fitness reaching significance at POz [β = 0.21, *p =* .05]. Other site-specific slopes were in the same direction but did not reach statistical significance (*p*’s > 0.05). Estimated marginal means are adjusted for sex, pubertal status, and mother’s education. Error bars represent ± 1 SE. *p < .05, ** *p <* .01, *** *p <* .001.

There was no main effect of Fitness on P3 amplitude, *F (*1, 96) = 0.82, *p =* .368; however as expected, there was a main effect of Electrode Site, *F (*6, 600) = 58.91, *p <* .001. The Fitness x Electrode Site interaction was also significant, *F (*6, 600) = 2.91, *p =* .008. Probing of EMMs revealed that higher-fit children showed larger P3 amplitudes at central, parietal and occipital sites (Cz, CPz, Pz, POz, and Oz), whereas differences between fitness levels and P3 amplitudes were smaller at frontal sites (Fz, FCz). See Table 2. Post hoc probing of simple slopes indicated that the fitness slope reached significance at POz (β = 0.21, *p =* .05), with slopes at remaining sites trending in the same direction but not reaching significance (all p’s > 0.05; see Fig. 2).

**Table 2:** P3 Amplitude EMMs by fitness.

Task	Site	Fitness + 1 SD			Fitness − 1 SD		
		95% CI	Mean	SE	95% CI	Mean	SE
Go	Fz	6.20, 11.59	8.90	1.37	7.53, 13.02	10.28	1.39
	FCz	7.65, 13.04	10.34	1.37	7.78, 13.26	10.52	1.39
	Cz	14.91, 20.30	17.60	1.37	13.19, 18.68	15.93	1.39
	CPz	17.15, 22.55	19.85	1.37	15.11, 20.59	17.85	1.39
	Pz	18.26, 23.65	20.95	1.37	15.29, 20.77	18.03	1.39
	POz	17.96, 23.36	20.66	1.37	14.02, 19.51	16.77	1.39
	Oz	15.08, 20.48	17.78	1.37	12.67, 18.15	15.41	1.39
NoGo	Fz	7.19, 12.05	9.62	1.23	8.74, 13.67	11.20	1.25
	FCz	8.32, 13.17	10.75	1.23	9.76, 14.70	12.23	1.25
	Cz	11.57, 16.43	14.00	1.23	13.04, 17.98	15.51	1.25
	CPz	12.10, 16.96	14.53	1.23	13.41, 18.35	15.88	1.25
	Pz	12.78, 17.64	15.21	1.23	12.51, 17.44	14.97	1.25
	POz	13.34, 18.19	15.76	1.23	12.78, 17.72	15.25	1.25
	Oz	13.58, 18.44	16.01	1.23	12.15, 17.09	14.62	1.25

Note. Estimated marginal means are shown at -1 SD (60.66 (mL/kg FFM/min) and + 1 SD (79.11 (mL/kg FFM/min) of fat-free peak oxygen consumption.

There was no main effect of Body Composition on P3 amplitude, *F (*1, 96) = 0.81, *p =* .373; however as expected, there was a main effect of Electrode Site, *F (*6, 600) = 2.77, *p =* .011. Additionally, there was no Body Composition x Electrode Site interaction *F (*6, 600) = 0.50, *p =* .812.

#### P3 Latency

The model revealed no main effect of Fitness on P3 latency, *F (*1, 96) = 0.03, *p =* .868; however as expected, there was a main effect of Electrode Site, *F (*6, 600) = 82.16, *p <* .001. The Fitness x Electrode Site interaction was not significant, *F (*6, 600) = 0.92, *p =* .484.

There was no main effect of Body Composition on P3 latency, *F (*1, 96) = 1.02, *p =* .315, but there was a main effect of Electrode Site, *F (*6, 600) = 5.01, *p <* .001. The interaction between Body Composition x Electrode Site was not significant, *F (*6, 600) = 0.99, *p =* .434.

### NoGo task

#### N2 Amplitude

**Fig. 3:**
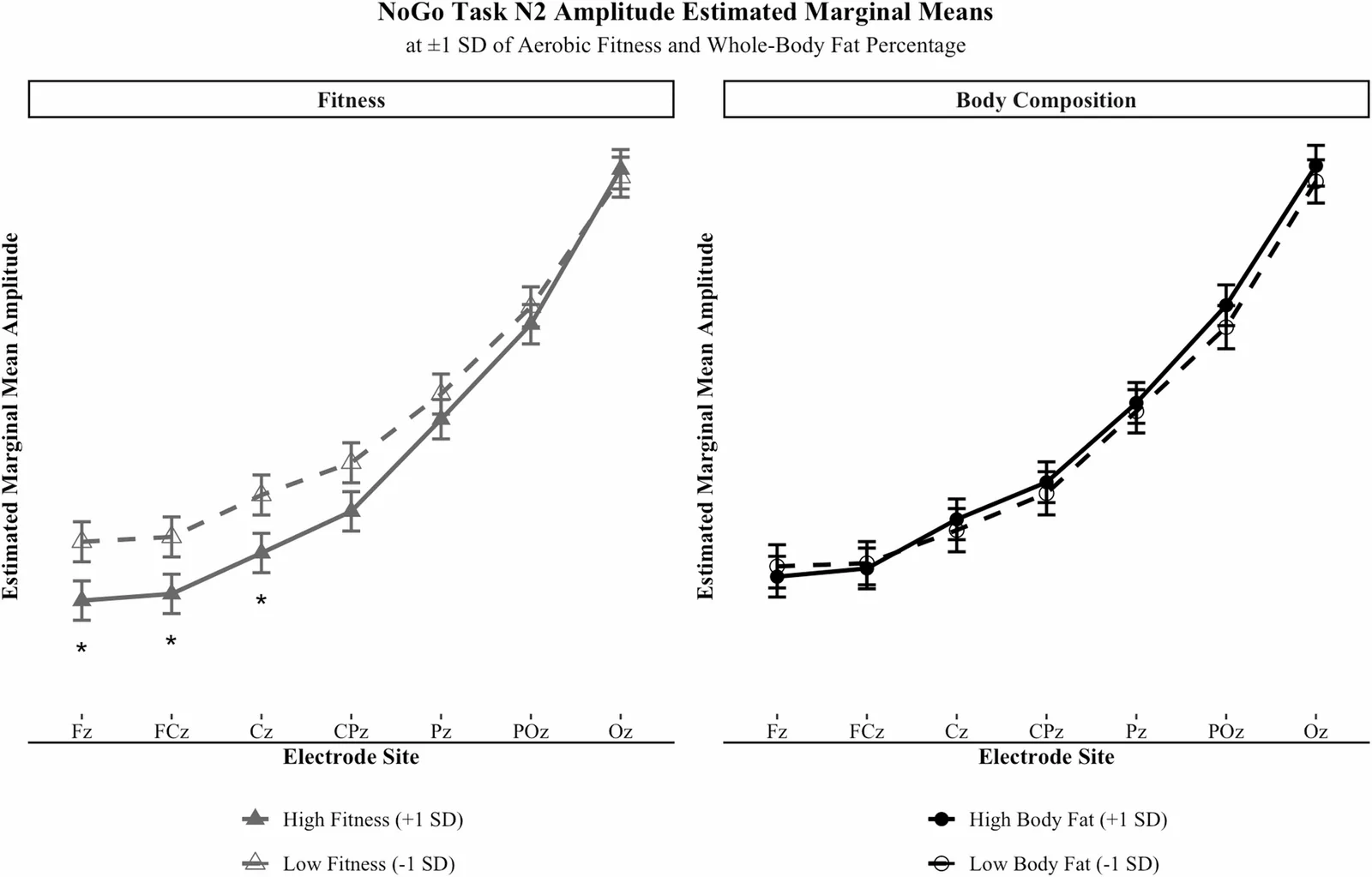
NoGo Task N2 Amplitude Estimated Marginal Means at ± 1 SD of Aerobic Fitness and Whole-Body Fat Percentage Across Seven Midline Electrode Sites. *Note.* A significant Fitness × Electrode Site interaction was observed *F (*6, 600) = 2.30, *p =* .033, reflecting a topographic pattern whereby higher fitness was associated with more negative N2 amplitudes at fronto-central sites. No significant Body Composition × Electrode Site effects were observed at individual electrode sites based on simple slopes probing (*p >* .05). Estimated marginal means are adjusted for sex, pubertal status, and mother’s education. Error bars represent ± 1 SE. * *p <* .05, ** *p <* .01, *** *p <* .001.

There was no main effect of Fitness on N2 amplitude, *F (*1, 96) = 2.45, *p =* .121, however as expected there was a main effect of Electrode Site, *F (*6, 600) = 299.50, *p <* .001. The Fitness x Electrode Site interaction was also significant, *F (*6, 600) = 2.30, *p =* .033. Probing of EMMs showed that higher fitness was associated with more negative N2 amplitudes at fronto-central electrode sites (Fz, FCz, Cz, CPz). This association became smaller and less negative over parietal sites (Pz, POz), and positive going at Oz. See Table 1. Post hoc probing of simple slopes revealed that the fitness slope was significant at Fz (β = -0.26, *p =* .039), FCz (β = -0.25, *p =* .046), and Cz (β = -0.25, *p =* .042), indicating that the fitness-N2 association was most pronounced at fronto-central electrode sites (see Fig. 3).

There was no main effect of Body Composition on N2 amplitude, *F (*1, 96) = 0.13, *p =* .721, but there was a main effect of Electrode Site, *F (*6, 600) = 11.12, *p <* .001. The interaction between ***Body Composition*** x Electrode Site was non-significant, *F (*6, 600) = 0.41, *p =* .872.

#### N2 Latency

There was no main effect of Fitness on NoGo N2 latency, *F (*1, 96) = 0.001, *p =* .984, however as expected there was a main effect of Electrode Site, *F (*6, 600) = 3.95, *p =* .001. The Fitness x Electrode Site interaction was not significant, *F (*6, 600) = 1.27, *p =* .271.

There was no main effect of Body Composition, *F (*1, 96) = 0.72, *p =* .397, nor Electrode Site *F (*6, 600) = 0.66, *p =* .681 on N2 latency. There was also no significant interaction between Body Composition x Electrode Site, *F (*6, 600) = 0.79, *p =* .578.

#### P3 Amplitude

There was no main effect of Fitness on P3 amplitude, *F (*1, 96) = 0.18, *p =* .669, however as expected there was a main effect of Electrode Site, *F (*6, 600) = 15.91, *p <* .001. The Fitness x Electrode Site interaction was not significant, *F (*6, 600) = 1.36, *p =* .230.

There was no main effect of Body Composition on P3 amplitude, *F (*1, 96) = 1.74, *p =* .190, but there was a main effect of Electrode Site, *F (*6, 600) = 2.30, *p =* .033. The Body Composition x Electrode Site interaction was non-significant *F (*6, 600) = 0.93, *p =* .477.

#### P3 Latency

There was no main effect of fitness on P3 latency, *F (*1, 96) = 2.46, *p =* .120, however as expected there was a main effect of Electrode Site, *F (*6, 600) = 52.05, *p <* .001. The Fitness x Electrode Site interaction was not significant, *F (*6, 600) = 1.16, *p =* .324.

There was no main effect of Body Composition on P3 latency, *F (*1, 96) = 0.69, *p =* .408, but there was a main effect of Electrode Site, *F (*6, 600) = 8.23, *p <* .001. There was a significant Body Composition x Electrode Site interaction, *F (*6, 600) = 2.17, *p =* .045. Based on the inspection of EMMs, higher percent body fat was associated with shorter latency along all midline electrode sites. See Table 3. However, post hoc probing of simple slopes indicated that no individual electrode sites reached significance (all p’s > 0.05), suggesting the interaction reflects a broad, distributed pattern of shorter latencies associated with higher percent body fat across the scalp rather than a focal effect at any single site.

**Table 3:** P3 Latency EMMs by whole body fat percent.

Task	Site	Whole-Body Fat % +1 SD			Whole-Body Fat % -1 SD		
		95% CI	Mean	SE	95% CI	Mean	SE
Go	Fz	646.69, 699.72	673.21	13.48	679.78, 736.31	708.05	14.36
	FCz	644.57, 697.60	671.08	13.48	652.30, 708.83	680.56	14.36
	Cz	619.23, 672.26	645.74	13.48	627.26, 683.79	655.52	14.36
	CPz	612.92, 665.94	639.43	13.48	623.61, 680.14	651.88	14.36
	Pz	600.17, 653.19	626.68	13.48	592.66, 649.19	620.93	14.36
	POz	529.18, 582.21	555.69	13.48	561.89, 618.42	590.15	14.36
	Oz	468.13, 521.16	494.64	13.48	478.58, 535.11	506.85	14.36
NoGo	Fz	645.09, 700.47	672.78	14.08	678.65, 737.63	708.14	14.99
	FCz	630.30, 685.68	657.99	14.08	662.37, 721.35	691.86	14.99
	Cz	627.80, 683.18	655.49	14.08	646.89, 705.87	676.38	14.99
	CPz	609.42, 664.80	637.11	14.08	634.39, 693.37	663.88	14.99
	Pz	596.38, 651.76	624.07	14.08	583.81, 642.79	613.30	14.99
	POz	570.12, 625.50	597.81	14.08	545.71, 604.70	575.20	14.99
	Oz	501.41, 556.78	529.10	14.08	495.53, 554.51	525.02	14.99

Note. Estimated marginal means are shown at -1 SD (21.25%) and + 1 SD (33.94%) of whole-body fat percent.

## Discussion

The present study examined the independent influences of aerobic fitness (FFVO_2_peak) and body composition (whole body fat percentage) on behavioral and neuroelectric indices of inhibitory control in preadolescent children. With respect to aerobic fitness, higher FFVO_2_peak was associated with better NoGo task accuracy but was not related to Go task behavioral performance. In support of this finding, fitness also showed site-specific interactions with N2 amplitude during both Go and NoGo tasks, with higher-fit children exhibiting more negative amplitudes at fronto-central sites. Further, fitness differentiated P3 amplitude during the Go task, with larger amplitudes at central-parietal and parietal-occipital sites in higher-fit children. With respect to body composition, higher whole body fat percentage was not related to accuracy on either task. Body composition did not interact with Go N2 or P3 amplitude but did show a significant relationship with NoGo P3 latency, such that higher body fat was associated with shorter latencies along the midline of the scalp. Taken together, these findings suggest that aerobic fitness and body composition are each associated with distinct patterns of neurocognitive performance and behavioral outcomes during inhibitory control.

The present findings partially support our hypothesis that higher FFVO_2_peak would be associated with superior behavioral performance and more effective neural processing during inhibitory control. At the behavioral level, higher fitness was associated with greater NoGo accuracy, but no significant associations were observed for Go task performance. This pattern of results is consistent with prior work demonstrating fitness-related advantages in inhibitory control among preadolescent children^9,11,12,14,15^ and suggests that fitness-related differences in cognitive performance are more apparent under conditions that place greater demands on inhibitory control, such as the NoGo task, requiring active suppression of a prepotent response, rather than under conditions of simple stimulus detection. This interpretation aligns with the hypothesis that the relationship between fitness and inhibition may be specific to situations producing increased cognitive conflict^37^.

At the neuroelectric level, no main effects of fitness on N2 or P3 amplitude or latency were observed for either task, which is consistent with prior research suggesting that fitness-related neuroelectric differences are not globally observed across the scalp, but rather are regional (i.e., site) and condition-specific^36,37^. In support of this, significant Fitness x Site relationships were found for N2 amplitude during both the Go and NoGo tasks, with higher-fit children exhibiting more negative N2 amplitudes at fronto-central sites (Fz, FCz, Cz, CPz), an effect that diminished over parietal sites and reversed in polarity at occipital sites. Given that the N2 component reflects inhibitory neural processing and conflict monitoring^32,35^, this fronto-central enhancement in higher-fit children suggests that greater aerobic fitness is associated with more effective engagement of the neural systems underlying response inhibition and conflict detection.

Further, a significant Fitness x Site effect was observed for P3 amplitude during the Go task, with higher-fit children showing larger amplitudes at central-parietal and parietal-occipital sites (Cz, CPz, Pz, POz, Oz) compared to lower-fit children. As P3 amplitude has been theorized to reflect the allocation of attentional resources during stimulus processing^25,40^, this finding suggests that higher-fit children may allocate greater neural resources during cognitive task performance, consistent with prior work linking aerobic fitness to enhanced P3 amplitude during tasks requiring inhibitory control^8,11,36^. Importantly, this fitness-related enhancement was regionally specific, with higher-fit children showing larger P3 amplitudes at posterior central and parietal sites rather than a global increase across the scalp. This posterior distribution is consistent with the typical Go task P3 topography^27,29^ and may suggest that higher-fit children engage the canonical attentional resource network more robustly during stimulus processing, reflecting a more typical and potentially more effective information processing stream. Notably, this same fitness effect was not observed during the NoGo task, which may reflect the possibility that the heightened inhibitory demands of the NoGo task introduced a sufficient cognitive load to reduce the sensitivity of P3 amplitude as a differentiating measure between fitness levels, as greater task demands have been shown to attenuate individual difference effects on attentional resource allocation^25,36^.

With regard to body composition, the present findings provided only limited support for our hypothesis that higher whole body fat percentage would be associated with poorer behavioral performance and less effective neural processing during inhibitory control. At the behavioral level, no significant associations were observed for either task. A ceiling effect in task performance was observed, which limited the sensitivity of accuracy as a dependent measure. Body composition did not interact with N2 or P3 amplitude at any electrode site for either task, which contrasts with prior findings showing body composition-related differences in ERP topography, including reduced N2 amplitude and altered P3 topographic distribution in children with obesity relative to healthy-weight peers^6,38^. The absence of amplitude interactions in the present study may reflect the use of a more precise DXA-derived fat percentage rather than BMI-based weight status categories. Notably, reliance on BMI alone would have obscured meaningful variability in this sample, as the majority of children would have been classified as healthy weight, giving the appearance of a largely homogeneous sample. DXA-derived body fat percentage revealed a normal distribution ranging from low to high adiposity (16.15% to 44.47%, *M* = 27.59%, *SD* = 6.31%), indicating that the limited body composition findings are unlikely to reflect a simple restriction of range. Rather, they may reflect a genuine absence of body composition-related differences in inhibitory control at the levels of adiposity represented here, consistent with prior work in which significant effects were observed primarily in samples with greater proportions of children with obesity^6,38^. However, significant Body Composition x Site effects were observed for NoGo P3 latency, with higher body fat associated with shorter latencies across all midline electrode sites. As P3 latency is considered an index of stimulus evaluation and processing speed^25^, this finding may indicate differences in the speed of cognitive processing in children with higher body fat. The physiological consequences of excess adiposity for executive function networks offer one interpretation of this pattern. Higher adiposity is associated with chronic low-grade systemic inflammation and with structural alterations in prefrontal and striatal regions and has been linked to executive function and inhibitory control deficits in children and adults^41^. Because adiposity is more typically linked to poorer and slower executive function^41^, the co-occurrence in the present sample of shorter latencies without any accompanying gain in accuracy, is unlikely to represent more efficient processing; it may instead reflect a tendency toward faster but less regulated responding, consistent with evidence linking higher adiposity to greater impulsivity in children^42^. Shorter latency is therefore not inherently indicative of more efficient inhibitory processing and, in the absence of corresponding behavioral differences, is difficult to interpret as reflecting a genuine advantage in inhibitory control^6,42^.

This study’s approach to measuring aerobic fitness and body composition are notable strengths. First, the simultaneous examination of aerobic fitness and body composition as independent predictors allowed for a more nuanced characterization of their respective contributions to inhibitory control, rather than treating them as interchangeable or conflated constructs. The use of DXA-derived whole body fat percentage represents a methodological advancement over prior work relying on BMI as a proxy for adiposity^6,19^. Because BMI does not distinguish between fat mass and lean mass, it provides an imprecise estimate of adiposity. The use of DXA allowed for a more direct and precise characterization of body composition, strengthening the interpretability of the observed associations with inhibitory control. Complementing this, the use of FFVO_2_peak as the fitness metric further reduces the potential for confounding by statistically accounting for individual differences in body composition, providing a more independent estimate of aerobic fitness. Together, these measurement choices strengthen the validity of the independent associations observed and support the interpretation that aerobic fitness and body composition exert distinct influences on inhibitory control.

Nevertheless, several limitations of the present study should be noted when interpreting these findings. First, the cross-sectional design precludes any causal inferences about the relationships between aerobic fitness, body composition, and inhibitory control. Longitudinal or experimental designs, such as exercise intervention studies, would be necessary to establish directionality and causality in these associations. Relatedly, the present analyses utilized only baseline data from the SNEACY trial, leaving open the question of whether changes in fitness or body composition over time are associated with corresponding changes in neurocognitive performance. Future work leveraging the longitudinal nature of the full dataset would help address this question. Second, the fixed task order, with the Go task always administered before the NoGo task, was intentional to build a prepotent response prior to requiring its inhibition, but this design nonetheless introduces the possibility of order effects such as fatigue or practice that cannot be fully disentangled from task condition effects. Third, the observed ceiling effect in task accuracy likely reduced sensitivity to detect body composition-related differences in behavioral performance and may have similarly constrained fitness-related accuracy effects. Future studies should consider task designs that better distribute performance across the full accuracy range to avoid this issue. Finally, the sample was recruited from the Greater Boston, MA region and was predominantly white and non-diverse, limiting the generalizability of these findings to broader and more demographically representative populations of preadolescent children. Future research should prioritize more diverse samples to improve the external validity of cognition, fitness, and body composition associations.

The present findings extend our understanding of the relationship between physical health and neurocognition in preadolescent children by revealing that aerobic fitness and body composition, while often correlated, contribute uniquely to the neurocognitive systems underlying inhibitory control. Each factor was associated with a distinct pattern of behavioral and neuroelectric effects, suggesting that their respective influences on cognitive development operate through separable pathways rather than a common mechanism. Higher aerobic fitness was associated with more accurate NoGo performance and enhanced neural indices of conflict monitoring and attentional resource allocation, whereas higher body fat was associated with shorter NoGo P3 latency in the absence of clear behavioral differences, a pattern that warrants further investigation. These dissociable effects have implications for the design of targeted interventions aimed at supporting neurocognitive health during this critical period of executive function development. Specifically, efforts to promote aerobic fitness and address excess adiposity may present unique and complementary benefits to the developing cognitive system, underscoring the value of considering both factors in school-based health and wellness programs. Future research that leverages longitudinal and experimental designs will be essential to establish whether targeted improvements in fitness or reductions in adiposity produce corresponding neurocognitive benefits and how those changes can be harnessed to support healthy cognitive development in children.

## Methods

### Participants

This dataset includes a sample of cognitively normal children between 9 and 11 years old who were recruited to participate in the SNEACY research trial in the Greater Boston, MA region (ClinicalTrials.gov Identifier number: NCT03592238). Study procedures were reviewed and approved by the Northeastern University Institutional Review Board and were performed in accordance with the Declaration of Helsinki. All participants (*N* = 116) provided written informed assent, and their legal guardians provided written informed consent. Participants with missing data for any of the primary measures (DXA, ERP, or VO2peak) were excluded, resulting in a final sample of 102 participants. See Table 4 for participant characteristics.

**Table 4:** Participant Characteristics.

FFVO_2_peak (mL/kg FFM/min)	*n*	M	S.E.
	102	69.88	0.88
Whole-body fat *(*%)	102	27.59	0.61
Pubertal Stage	94	1.53	0.06

	*n*	%	
Sex			
Female	48	47	
Male	54	53	
Mother’s education			
High school graduate	2	2	
Some college	16	15.7	
Bachelor’s degree	33	32.3	
Advanced degree	51	50	

Note. FFVO_2_peak = fat-free peak oxygen consumption. Pubertal stage was assessed via the Tanner staging criteria; *n* = 94 due to missing data. Percentages for sex are based on valid responses (*n* = 102). SE = standard error.

### Laboratory procedures

During the first visit, participants and their legal guardians completed a detailed health and demographics questionnaire, a pre-participation health screening questionnaire, and the modified Tanner pubertal self-assessment questionnaire^43^. Participants then completed a Kaufman Brief Intelligence Test, Second Edition (KBIT-2;^44^ before undergoing body composition measurements. The session concluded with a maximal cardiorespiratory fitness assessment. During the second visit, participants were outfitted with a Neuroscan Quik-cap (Compumedics, Inc., Charlotte, NC) and performed a Go/NoGo task in front of a computer screen while EEG data were simultaneously recorded. Although participants repeated this task during the third and fourth sessions, only baseline data from the second visit were utilized in the present analysis.

### Body composition

Participants were instructed to wear or change into lightweight, metal-free clothing. Height and weight were measured using a Tanita WB-300 Plus digital scale (Tanita Corp., Tokyo, Japan), and body composition was assessed via dual-energy X-ray absorptiometry (DXA; GE Lunar iDXA, Madison, WI). Whole body fat percentage was calculated as total body fat mass divided by total body mass.

### Cardiorespiratory fitness (CRF)

At the end of their first session, participants completed a VO_2_peak test on a motorized treadmill (Treadmill: Trackmaster TMX428; Metabolic Cart: COSMED Quark CPET OMNIA, Concord, CA) using a modified Balke protocol based on recommendations from the American College of Sports Medicine (2018). After a brief warm-up, children ran or walked at a constant speed with incremental intensity increases via grade inclines of 2.5% every 2 min until volitional fatigue. Ratings of perceived exertion (RPE) were assessed every two minutes using the children’s OMNI scale^45^. Maximal effort was determined by: (1) a plateau in oxygen consumption corresponding to an increase of less than 2 ml/kg/min despite an increase in workload, (2) a peak heart rate (HR) ≥ 185 bpm^46^ and a plateau in HR, (3) a respiratory exchange ratio (RER) ≥ 1.0^47^, and/or (4) ratings on the children’s OMNI scale of perceived exertion ≥ 8^45^. FFVO_2_peak was calculated as absolute VO_2_peak divided by total body lean mass (excluding the head).

### Go/NoGo task

During the second visit, children performed a Go/NoGo task while wearing an EEG cap. The task was presented, and responses were recorded, using E-prime software (Psychology Software Tools, Inc., Sharpsburg, PA). In the Go task, participants were instructed to press a button with their right thumb in response to rare target stimuli (20% probability, a simple illustration of a lion), displayed within a sequence of frequent non-target stimuli (80% probability, a simple illustration of a tiger), which did not require a response. Following the Go task, participants then completed the NoGo task, in which they were instructed to press a button in response to the frequent non-target stimuli (80% probability, tiger) and withhold their response to the rare target stimuli (20% probability, lion). The fixed task order was used to build a prepotent response to the rare stimuli before requiring inhibition.

Participants were asked to respond as quickly and accurately as possible. The experimenter provided instructions before each condition, and 20 practice trials were administered prior to testing. Participants’ comprehension of the task was verified during practice trials to ensure understanding and task mastery of greater than 50%. Participants completed one block of 250 trials for each task (Go and NoGo). Stimulus duration was 200 ms with a 1,500 ms intertrial interval. Response accuracy for the Go task was defined as the proportion of correct responses for the rare target stimuli, and for the NoGo task as the proportion of correct withheld responses for the rare target stimuli. Reaction time (RT) for the Go task was defined as the mean reaction time for trials in which the response was correct based on the task instructions. Standard deviation of reaction time (SDRT) for the Go task was calculated as the standard deviation of the reaction time for correct responses.

### ERP recording and reduction

Neuroelectric activity was measured from 64-electrode sites and 3 integrated bipolar leads for vertical and horizontal eye movements (VEOG, HEOG) in a configuration consistent with the 10–10 system using a Neuroscan Quik-cap (Compumedics, Inc., Charlotte, NC). Electrodes were placed above and below the right orbit and on the outer canthus of each eye to record bipolar eye movements. Data were continuously digitized at 1,000 Hz, amplified 500 times, and filtered with a DC to 70 Hz filter and a 60 Hz notch filter using a Neuroscan Neuvo amplifier (Compumedics, Inc., Charlotte, NC). Additional offline processing was performed using MATLAB (Version 2021b, MathWorks, Inc.), open source EEGLab (Version 14.1.2b;^48^, and ERPLab (Version 7.0;^49^. Data were re-referenced offline to average mastoids (M1 and M2). Continuous data were corrected for EOG artifacts using a spatial filter. Stimulus-locked epochs were created for trials (-200 to 1,000 ms relative to stimulus onset) and filtered using a causal Butterworth low pass filter (30 Hz, 24 dB/octave). Afterwards, an independent component analysis (i.e., ICA) was performed and eyeblink artifacts were identified and removed according to an autocorrelation function called “icablinkmetrics” using VEOG activity^50^. Bad channels were then interpolated using spherical interpolation. Trials with artifacts that exceeded ± 75 µV were rejected. Grand averaged ERP waveforms were created for each condition and stimulus, and components were calculated from these grand averages. The N2 (200–400 ms) and P3 (300–850 ms) time windows were selected a priori based on prior literature^25,30,31^ and confirmed through visual inspection of the grand average waveforms in the present sample. The N2 component was assessed as the largest negative-going peak within the 200–400 ms latency window. The P3 component was assessed as the largest positive-going peak within the 300–850 ms latency window. Amplitude was measured as the difference between the mean pre-stimulus baseline and mean amplitude within a 50 ms window centered on the peak latency (-25 to + 25 ms); peak latency was defined as the time point corresponding to the maximum amplitude.

### Statistical analysis

All analyses were conducted in IBM SPSS Statistics (Version 28, SPSS Inc., Chicago, IL) with a primary significance level set at *p =* .05.

Associations between aerobic fitness (FFVO_2_peak, z-scored), body composition (body percent fat) and behavioral performance were examined using multiple linear regression models using Bonferroni correction to control for multiple comparisons. Separate models were conducted for the Go and NoGo tasks, with accuracy as the dependent variable. Each model included fitness or body composition as a continuous predictor, with the other included as a covariate along with sex, mother’s education, and Tanner pubertal status^51^.

For ERP outcomes, linear mixed-effects models were used to investigate associations between aerobic fitness and ERP amplitudes and latencies across scalp sites. The N2 and P3 components were analyzed separately for the Go and NoGo tasks. Each model included fitness (z-scored) or body composition, site (Fz, FCz, Cz, CPz, Pz, POz, Oz), sex, mother’s education, and pubertal status as fixed effects, with a random intercept for participant to account for repeated measures across sites using Bonferroni correction to control for multiple comparisons. Within-subject correlations among electrode sites were specified as a compound symmetry covariance structure in the model. For significant Fitness x Site or Body Composition x Site interactions, model-based estimated marginal means (EMMs) were probed at -1 SD and + 1 SD of each predictor (z-scored for fitness; raw mean values at -1/+1 SD for body composition) to describe how the association between fitness and ERP varied across electrode sites.

## Supplementary Information

Below is the link to the electronic supplementary material.

Supplementary Material 1

## Data Availability

The datasets generated during and/or analyzed during the current study are not publicly available due to restrictions involving participant privacy but are available from the corresponding author upon reasonable request and with approval from the IRB. Requests should be submitted to c.hillman@northeastern.edu.
