# Supplementary material for: Aerobic fitness and body composition independently relate to inhibitory control in preadolescent children
Source: Sci Rep. 2026 Jul 18;16:32820. doi: 10.1038/s41598-026-63026-9 (PMC13677439; doi:10.1038/s41598-026-63026-9)
Supplement: Supplementary file 1 [file 41598_2026_63026_MOESM1_ESM.docx]

**Supplemental**

**Three-Way Interactions: Fitness x Body Composition x Electrode Site**

The primary analyses examined the independent contributions of fitness and body composition to ERP amplitude and latency by entering both predictors simultaneously in linear mixed-effects models, thereby statistically controlling for the shared variance between them. To further explore whether the relationship between fitness and ERP components varied as a function of body composition, and vice versa, a supplemental set of models was run that additionally included the three-way Body Composition x Fitness x Electrode Site interaction term.

These three-way models are not necessarily superior to the primary analyses. Because both predictors were already entered simultaneously in the primary models, each was already accounting for the other's contribution. The three-way models add complexity by estimating an additional interaction term, which redistributes variance across more parameters and reduces the power to detect the independent effects of each predictor. These independent effects were the primary focus of the study's research questions. Consistent with this, several fitness- and body composition-related interactions that emerged in the primary analyses (e.g., No-Go N2 Fitness x Electrode Site, Go P3 Fitness x Electrode Site) were attenuated in the three-way models, likely reflecting this redistribution of variance rather than a meaningful change in the underlying relationships. The three-way models are therefore presented here as exploratory supplemental analyses, and the primary two-way results remain the more appropriate framework for addressing the study's central research questions.

**Go Task**

***N2 Amplitude***

The three-way linear mixed-effects model revealed no main effect of Fitness, F (1, 95) = 1.10, p = .296, no main effect of Body Composition, F (1, 95) = 0.49, p = .487, but as expected, a significant main effect of Electrode Site, F (6, 588) = 21.11, p < .001. The Fitness x Electrode Site interaction was not significant, F (6, 588) = 0.71, p = .641, nor was the Body Composition x Electrode Site interaction, F (6, 588) = 0.14, p = .992. The three-way Body Composition x Fitness x Electrode Site interaction was also non-significant, F (7, 335.47) = 1.36, p = .221.

***N2 Latency***

There was no main effect of Fitness, F (1, 95) = 1.13, p = .291, no main effect of Body Composition, F (1, 95) = 0.87, p = .352, and no main effect of Electrode Site, F (6, 588) = 0.77, p = .593. The Fitness x Electrode Site interaction was not significant, F (6, 588) = 0.41, p = .876, nor was the Body Composition x Electrode Site interaction, F (6, 588) = 0.62, p = .713. The three-way Body Composition x Fitness x Electrode Site interaction was also non-significant, F (7, 432.28) = 0.51, p = .827.

***P3 Amplitude***

There was no main effect of Fitness, F (1, 95) = 0.12, p = .731, no main effect of Body Composition, F (1, 95) = 0.75, p = .388, but as expected, a significant main effect of Electrode Site, F (6, 588) = 2.33, p = .032. The Fitness x Electrode Site interaction was not significant, F (6, 588) = 0.80, p = .571, nor was the Body Composition x Electrode Site interaction, F (6, 588) = 0.34, p = .916. The three-way Body Composition x Fitness x Electrode Site interaction was also non-significant, F (7, 335.47) = 0.72, p = .659.

***P3 Latency***

There was no main effect of Fitness, F (1, 95) = 3.93, p = .050, no main effect of Body Composition, F (1, 95) = 0.66, p = .419, but as expected, a significant main effect of Electrode Site, F (6, 588) = 5.09, p < .001. The Fitness x Electrode Site interaction was not significant, F (6, 588) = 0.76, p = .605, nor was the Body Composition x Electrode Site interaction, F (6, 588) = 0.77, p = .592. The three-way Body Composition x Fitness x Electrode Site interaction was also non-significant, F (7, 324.22) = 1.30, p = .247.

**No-Go Task**

***N2 Amplitude***

The three-way linear mixed-effects model revealed no main effect of Fitness, F (1, 95) = 0.60, p = .440, no main effect of Body Composition, F (1, 95) = 0.06, p = .810, but as expected, a significant main effect of Electrode Site, F (6, 588) = 9.03, p < .001. The Fitness x Electrode Site interaction was not significant, F (6, 588) = 1.02, p = .410, nor was the Body Composition x Electrode Site interaction, F (6, 588) = 0.75, p = .608. The three-way Body Composition x Fitness x Electrode Site interaction was also non-significant, F (7, 335.47) = 1.63, p = .127.

***N2 Latency***

There was no main effect of Fitness, F (1, 95) = 1.69, p = .197, no main effect of Body Composition, F (1, 95) = 0.50, p = .483, and no main effect of Electrode Site, F (6, 588) = 0.44, p = .855. The Fitness x Electrode Site interaction was not significant, F (6, 588) = 0.86, p = .527, nor was the Body Composition x Electrode Site interaction, F (6, 588) = 0.50, p = .813. The three-way Body Composition x Fitness x Electrode Site interaction was also non-significant, F (7, 357.04) = 0.94, p = .478.

***P3 Amplitude***

There was no main effect of Fitness, F (1, 95) = 0.01, p = .914, no main effect of Body Composition, F (1, 95) = 1.72, p = .194, and no significant main effect of Electrode Site, F (6, 588) = 1.72, p = .113. The Fitness x Electrode Site interaction was not significant, F (6, 588) = 0.81, p = .561, nor was the Body Composition x Electrode Site interaction, F (6, 588) = 0.63, p = .705. The three-way Body Composition x Fitness x Electrode Site interaction was also non-significant, F (7, 335.47) = 0.99, p = .439.

***P3 Latency***

There was no main effect of Fitness, F (1, 95) = 3.75, p = .056, no main effect of Body Composition, F (1, 95) = 0.36, p = .548, but as expected, a significant main effect of Electrode Site, F (6, 588) = 6.95, p < .001. The Fitness x Electrode Site interaction was not significant, F (6, 588) = 1.35, p = .232, nor was the Body Composition x Electrode Site interaction, F (6, 588) = 1.70, p = .119. The three-way Body Composition x Fitness x Electrode Site interaction was significant, F (7, 367.71) = 2.12, p = .041.
